# Ambipolar inverters with natural origin organic materials as gate dielectric and semiconducting layer

**DOI:** 10.1002/pssr.201510139

**Published:** 2015-06-11

**Authors:** Andreas Petritz, Alexander Fian, Eric D. Głowacki, Niyazi Serdar Sariciftci, Barbara Stadlober, Mihai Irimia‐Vladu

**Affiliations:** ^1^ Joanneum ResearchMaterials Institute for Surface Technologies and PhotonicsFranz‐Pichler Straße 308160WeizAustria; ^2^ Linz Institute for Organic Solar Cells (LIOS)Johannes Kepler UniversityAltenbergerstraße 694040LinzAustria

**Keywords:** organic complementary‐like inverters, ambipolar inverters, natural origin organic materials, nature‐inspired materials, organic electronics

## Abstract

Thin film electronics fabricated with non‐toxic and abundant materials are enabling for emerging bioelectronic technologies. Herein complementary‐like inverters comprising transistors using 6,6′‐dichloroindigo as the semiconductor and trimethylsilyl‐cellulose (TMSC) films on anodized aluminum as bilayer dielectric layer are demonstrated. The inverters operate both in the first and third quadrant, exhibiting a maximum static gain of 22 and a noise margin of 58% at a supply voltage of 14 V. (© 2015 WILEY‐VCH Verlag GmbH &Co. KGaA, Weinheim)

## 1 Introduction

Complementary metal‐oxide‐semiconductor (CMOS) circuits are a fundamental building block of integrated circuits (ICs). The realization of competitive organic semiconductor‐based ICs hinges upon creating complementary‐like logic elements with similar function and performance. The approach of using discrete n‐ and p‐type organic materials requires the necessity of their lateral patterning, making device fabrication on a common substrate a very complex process. Ambipolar organic semiconductors which provide both p‐ and n‐type operation enable complementary‐like circuits that minimize patterning and complex fabrication processes while still exhibiting reasonably high static gains 1, 2, 3, 4, 5. In one example, based on the ambipolar natural dye indigo semiconductor, commensurably high static gain values were demonstrated due to the balanced electron and hole mobility in this material 6, 7. Besides being doubtlessly better compatible with low‐cost fabrication, the draw‐back of ambipolar inverters is that they cannot be fully switched off 8, when one of the stable output voltages is reached. Thus, strict rail‐to‐rail operation is not possible, and therefore the noise margin is reduced compared to conventional complementary logic. Recent work has shown the possibility of employing natural semiconducting materials in ambipolar inverters 9. Inspired by the excellent performance of complementary organic inverters with TMSC/Al_2_O_3_ dielectrics and by the potential of hydrogen‐bonded nature‐inspired semiconductors we developed a complementary‐like inverter made up of two identical ambipolar OTFTs with 6,6′‐dichloroindigo as the organic semiconductor and the TMSC‐based bilayer as the gate dielectric. Both, trimethylsilyl cellulose and 6,6′‐dichloroindigo employed in this work are synthetic derivatives of the naturally occurring counterparts cellulose and indigo, the latter being derived from the hydrolysis of the freely occurring glycoside indican from Indigofera genus plants. These ambipolar inverters show a reasonable performance with gain values up to 22, however, accompanied by the typical lack of rail‐to‐rail operation and a slight asymmetry in the voltage transition curve that is due to the imbalanced electron and hole mobility values.

## 2 Experimental

### 2.1 Anodization of aluminum

A thin Al_2_O_3_ dielectric layer was grown electrochemically on a 60 nm thermal evaporated aluminum gate electrode, using a method reported by Lohrengel et al. and Mardare et al. 10, 11. The 60 nm thick aluminum gate electrode was thermal evaporated on a 1.5 × 1.5 cm^2^ glass slide at a rate of ∼0.5 nm/s and a typical pressure of ∼5 × 10^–6^ mbar.

Subsequently, the Al‐gate electrode was connected to the positive terminal of a Keithley 2401 low voltage source meter instrument and using a counter electrode of pure steel as the negative terminal is inserted partially in a citric acid solution (0.265 g citric acid, 2.57 g sodium citrate to give a pH of ∼6.0 in 100 ml, 18.2 MΩ water). The suspended portion of the gate electrode was used as gate contact for the electrical measurements. The surface of the immersed area of the Al electrode was anodized by passing a step voltage (up to a maximum of 20 V) at a constant current of 100 mA. The thickness of the anodized aluminum layer can be estimated from the applied anodization potential, considering the high field oxide growth factor of about 1.6 nm per each applied Volt for aluminum 11. Ellipsometry measurements indeed revealed a thickness of ∼28 nm for the electrochemically grown Al_2_O_3_. At the constant current of 100 mA, the 20 V compliance of anodization potential is reached almost instantaneously; however desired quality of aluminum oxide is obtained when the anodization current drops to a steady‐state value of about 0.5 µA for our geometry; this is typically achieved within 8–10 min from the start of anodization.

### 2.2 TMSC film preparation

**Figure 1:**
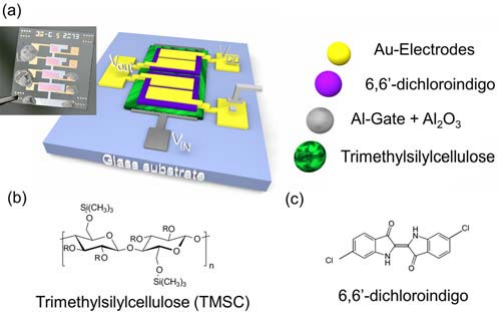
(a) Schematic image of an ambipolar inverter with TMSC (trimethylsilyl‐cellulose) as gate dielectric layer. In the inset photograph of two inverters on a glass slide are shown. Silver conductive ink was used to simplify the contacting. The chemical structures of TMSC and 6,6′‐dichloroindigo are displayed in (b) and (c), respectively. R in (b) stands for –Si(CH_3_)_3_ or –H depending on the degree of substitution.

TMSC, supplied by Thuringian Institute of Textile and Plastic Research with a degree of substitution (DS) of 2.8, was dissolved in chloroform and applied by spin coating, with a concentration of 5 mg/ml for 30 nm thin films. The chemical structure is shown in Fig. 1b. The layer thicknesses of TMSC films were determined by profilometer measurements (VEECO Dektak 150).

### 2.3 Device fabrication

Ambipolar organic thin film transistors (OTFTs) were fabricated in a staggered bottom‐gate, top contact architecture according to the layer setup illustrated in Fig. 1. The gate electrode was processed on a pre‐cleaned glass substrate by thermal evaporation of a 60 nm thick aluminum layer in a high vacuum system through a shadow mask at a rate of 0.5 nm/s, followed by anodization of the aluminum to create a 28 nm thick Al_2_O_3_. Next, an about 30 nm thick TMSC layer was spin coated followed by thermal evaporation of a 60 nm thick 6,6′‐dichloroindigo film. Source and drain electrodes were deposited by thermal evaporation of gold through a shadow mask in order to form 50 nm thick contacts. After production, all OTFT samples were protected from light and stored under argon atmosphere. The channel lengths of the OTFTs vary between 10 µm and 50 µm and the channel widths between 1 mm and 4 mm. The complemen‐ tary‐like inverters were fabricated by connecting two ambipolar OTFTs as shown in Fig. 1a. In the inset of Fig. 1a two inverters with TMSC as bilayer dielectric and 6,6′‐dichloroindigo as semiconductor are illustrated.

### 2.4 Electrical characterization

Electrical measurements of ambipolar OTFTs and complementary‐like inverters were carried out in a glove box under argon using a parameter analyzer from MB‐Technologies.

## 3 Results

The chemical structure of 6,6′‐dichloroindigo is depicted in Fig. 1c. This material was previously successfully integrated in organic thin film transistors, where bi‐layer dielectrics of anodized aluminum and tetratetracontane (C_44_H_90_) 12, or anodized aluminum and tetracontane (C_40_H_82_) were used 14. 6,6′‐dichloroindigo is a small band‐gap material, therefore providing electron and hole injection with only one appropriate electrode material. A HOMO energy level of –5.4 eV and a LUMO energy level of –3.6 eV are reported for evaporated thin‐films of 6,6′‐dichloroindigo 12. As gate dielectric a thin film of TMSC is used. The dielectric properties are reported in detail in Ref. 13. The channel length and channel width of the OTFTs devices are 25 µm and 2 mm, respectively.

**Table 1:** Parameters of the gate dielectric and fabricated transistors with Al_2_O_3_ + TMSC as gate dielectric, 6,6′‐dichloroindigo as the semiconductor and Au as source/drain electrodes

dielectric	*C* ′ (nF/cm^2^)^a)^		–*V*_on_ –(V)^b)^	–*V*_thr_ –(V)^c)^	*S* (V/dec.)^d)^	*µ*_sat_ (cm^2^/Vs)^e)^
28 nm Al_2_O_3_ + 30 nm TMSC	56 (±5)	n‐type	–4.6 (1.3)	–9.1 (1.1)	1.9 (0.5)	1.5 × 10^–2^ (0.7 × 10^–2^)
p‐type	–12.5 (0.7)	–14.2 (1.0)	1.8 (0.4)	0.9 × 10^–3^ (0.2 × 10^–3^)
						

**Figure 2:**
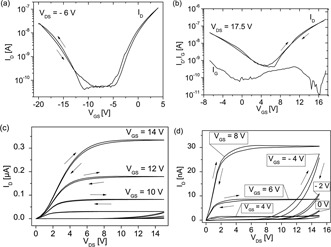
Transfer‐ (a, b) and output‐characteristics (c, d) of ambipolar OTFT with 6,6′‐dichloroindigo as semiconductor and TMSC on anodized Al as hybrid gate dielectric layer.

Transfer characteristics of 6,6′‐dichloroindigo based OTFTs are displayed in Fig. 2a, b, revealing a clear ambipolar charge transport for positive and negative source–drain voltages. The threshold voltages are *V*_thr, n_ = 8 V for n‐channel operation and *V*_thr, p_ = –14.1 V for p‐channel operation. In Fig. 2c, d the output characteristics of these OTFTs are plotted, showing ambipolar behavior. Figure 2d is a close‐up of *I*_DS_(*V*_DS_) for the small gate bias regime. The subthreshold swing values for n‐ and p‐type operation are very similar, between 1.8–1.9 V, and for the charge carrier mobility average values of about 1.5 × 10^–2^ (±0.7 × 10^–2^) cm^2^ V^–1^ s^–1^ for electrons and 0.9 × 10^–3^ (±0.2 × 10^–3^) cm^2^ V^–1^ s^–1^ for holes are extracted. These extracted mobilities are quite similar to values shown in Ref. 14. All OTFTs device parameters extracted from these curves are listed in Table 1.

**Figure 3:**
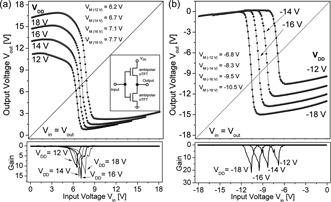
Voltage transfer characteristics (VTCs) with corresponding small‐signal gains of complementary‐like inverters for (a) positive and (b) negative supply voltages.

Two identical 6,6′‐dichloroindigo based OTFTs with the hybrid cellulose gate dielectric are integrated in a complementary‐like inverter. The inverter circuit is shown in the inset of Fig. 3a and two inverters on a glass slide are illustrated in the inset of Fig. 1a. The typical characteristics of such inverters (voltage transfer characteristics, VTCs) are shown in Fig. 3. Additionally to the VTCs the static gain is also plotted in the bottom lines of Fig. 3a and b for positive and negative supply voltages, respectively.

**Table 2:** Summary of complementary‐like inverter performance

–*V*_DD_–(V)^a)^	gain (V/V)^b)^	–*V*_M_–(V)^c)^	∆*V*_in_ (V)^d)^	NM_MEC_ (V)^e)^	NM_MEC_ (%)^f)^
–12	10	– 6.2	2.7	3.4	56
–14	15	– 6.7	2.2	4.1	58
–16	16	– 7.1	2.5	4.5	56
–18	14	– 7.7	–	4.7	52
–12	16	–6.8	0.8	3	51
–14	18	–8.3	1.0	3.3	48
–16	21	–9.5	1.1	3.6	45
–18	22	–10.5	1.1	3.7	41

The inverter worked well in both the first (VTC in Fig. 3a) and third quadrant (VTC in Fig. 3b) with a maximum noise margin of 58% and a maximum static gain value of 22 V/V. All inverter parameters extracted from these curves are listed in Table 2. As is typical for ambipolar inverters no exactly defined high and low levels are developed in the voltage transfer characteristics neither for positive nor for negative supply/input voltages.

## 4 Discussion

Ambipolar behavior is in principle an intrinsic property of all (undoped) organic semiconductors but in practice the presence of trap states at the interface (preferably for electrons) and the oxygen instability makes most semiconductors preferentially transport holes or electrons 15. Thus, complicated bi‐layer or heterostructure systems are often used to achieve ambipolarity, whereas in the chlorinated indigo‐based OTFTs reported here only one semiconductor layer is needed. Additionally, for an effective ambipolar performance typically asymmetric ontacts are used. A narrow band‐gap material like 6,6′‐dichloroindigo (band gap of 1.8 eV) 12, 14 has the big advantage of allowing n‐ and p‐type injection with the same electrode material (gold). Although electron and hole mobilities are not very high (10^–3^ cm^2^/V s to 10^–2^ cm^2^/V s), the distinct ambipolar nature of the chlorinated indigo‐based OTFTs is clearly demonstrated. The imbalance in the electron and hole mobilities results in a slight asymmetry of the VTC.

The big advantage of complementary‐like inverters based on ambipolar OTFTs is that they can be fabricated with a single type of transistor based on only one semiconductor and, as in the case of 6,6′‐dichloroindigo, with single contact material. In contrast, for the complementary pentacene/C_60_ inverters, apart from the use of two semiconductors, also two contact materials are needed for proper performance thus complicating the fabrication process on a single substrate. The inverters built up of two identical chlorinated‐indigo OTFTs work with reasonable static gains, however are impaired by a fundamental disadvantage of such complementary‐like inverters. In such devices the output voltage swing is limited (no full rail‐ to‐rail swing, 0 to *V*_DD_) corresponding to a lack of complete off states of both transistors, resulting in a strong reduction of the noise margin of the inverter compared to real complementary logic. This also heavily limits the applicability of such elements in more complex organic electronic circuits 8. Nevertheless, careful optimization of the processing of both the dielectric and the semiconductor material (scrupulous cleaning, improved deposition by identifying the optimum deposition parameters, i.e. spin coating speed and drying conditions for TMSC; vacuum deposition rate and substrate temperature for chlorinated indigo respectively; improved deposition of contact electrodes by finding the optimum deposition rate for a reduced contact resistance) may lead to well‐balanced n‐ and p‐channels of the individual transistors. This fact may improve the inverter parameters dramatically, as was shown already with other H‐bonded molecules tyrian purple, indigo and quinacridone 6, 16, 17.

## 5 Conclusion

Ambipolar OTFTs with 6,6′‐dichloroindigo as the semiconductor and cellulose based materials as gate dielectric are showing clear p‐ and n‐type operation. Two identical ambipolar OTFTs were integrated in a complementary like inverter, exhibiting a maximum static gain of 22 and a maximum noise margin of 58%. These ambipolar OTFTs represent an approach to high‐performance complementary‐like circuits that minimize patterning and complex fabrication processes with reasonably high static gains.
